# Clinical Impact of New Delhi Metallo-Beta-Lactamase-Producing Enterobacterales in Critically Ill Patients: Are We Ready to Face the Challenge?

**DOI:** 10.3390/jcm14165688

**Published:** 2025-08-12

**Authors:** Giorgia Montrucchio, Silvia Corcione, Lara Rodigari, Denisa Barganou, Chiara Risso, Riccardo Traversi, Gabriele Sales, Marco Ellena, Andrea Costamagna, Nour Shbaklo, Cecilia Grosso, Carlo Silvestre, Anna Chiara Trompeo, Vito Fanelli, Antonio Curtoni, Cristina Costa, Francesco Giuseppe De Rosa, Luca Brazzi

**Affiliations:** 1 Department of Surgical Sciences, University of Turin, 10126 Turin, Italy; lara.rodigari@unito.it (L.R.); denisa.barganou@edu.unito.it (D.B.); chiara.risso@unito.it (C.R.); riccardo.traversi@unito.it (R.T.); g.sales@unito.it (G.S.); andrea.costamagna@unito.it (A.C.); vito.fanelli@unito.it (V.F.); luca.brazzi@unito.it (L.B.); 2 Department of Anesthesia, Intensive Care and Emergency, Città della Salute e della Scienza University Hospital, 10126 Turin, Italy; mellena@cittadellasalute.to.it (M.E.); atrompeo@cittadellasalute.to.it (A.C.T.); 3 Department of Medical Sciences, University of Turin, 10126 Turin, Italy; silvia.corcione@unito.it (S.C.); nour.shbaklo@unito.it (N.S.); cecilia.grosso@unito.it (C.G.); francescogiuseppe.derosa@unito.it (F.G.D.R.); 4 Division of Geographic Medicine, Tufts University School of Medicine, Boston, MA 02111, USA; 5 S.C. Health Directorate Città della Salute e della Scienza University Hospital, 10126 Turin, Italy; carlo.silvestre@cittadellasalute.to.it; 6 Department of Public Health and Paediatrics, University of Turin, 10126 Turin, Italy; antonio.curtoni@unito.it (A.C.); cristina.costa@unito.it (C.C.); 7 Microbiology and Virology Laboratory, Città della Salute e della Scienza University Hospital, 10126 Turin, Italy

**Keywords:** New Delhi metallo-beta-lactamase, drug resistance, carbapenemase-producing Enterobacteriaceae, antimicrobial resistance, critical care, intensive care units, hospital infection control

## Abstract

**Background:** Carbapenem-resistant *Enterobacterales* infections are frequent in critically ill patients. Outbreaks caused by carbapenemase-producing *Enterobacterales*, in particular the New Delhi Metallo-beta-lactamase (NDM)-type carbapenemase-producing phenotype, are increasing in Italy. Unfortunately, the clinical impact of this new microorganism is still being defined, as well as the correlation between colonization and invasive infections. The aim of the study is to analyze factors related to the development of NDM infections in colonized patients and to evaluate their impact on patients’ outcome in high-complexity ICUs. **Methods:** All patients admitted to the General and Cardiac ICUs of ‘Città della Salute e della Scienza’ University Hospital in Turin (Italy) between January and August 2023 were enrolled. Microorganisms were examined by lateral flow immunochromatographic assays or molecular assays on weekly surveillance or clinically requested cultures. Antimicrobial susceptibility was determined by broth microdilution methods and interpreted according to EUCAST breakpoints. **Results:** Out of a total of 915 patients, 46 (5%) were positive for NDM-producing *Enterobacterales* and, among them, 13 (28%) developed an invasive infection. All patients were critical (SAPS II 40+/−13). The median times between ICU admission and colonization or infection were 6 and 16 days, respectively. Significant disparities emerged between colonized and infected patients regarding days of mechanical ventilation (1 vs. 28), ICU (7 vs. 39 days), and in-hospital (21 vs. 71 days) length of stay. Renal replacement treatment (OR 8.2461, *p* = 0.0173, 95% CI [1.3636–65.9114]) and surgery (OR 22.8747, *p* = 0.0149, CI95% [1.5986–1447.743]) seemed to impact the risk of developing infection. Six patients with invasive infection were treated with Cefiderocol and five with Ceftazidime/Avibactam and Aztreonam. In absence of early identification and appropriate treatment, patients may be at increased risk of colonization spread and potentially worse clinical outcomes. **Conclusions:** Early identification of the carbapenemase type is clinically relevant in critically ill patients with confirmed or suspected infection, as NDM production necessitates the use of specific agents for effective treatment.

## 1. Introduction

In critically ill patients admitted to the Intensive Care Unit (ICU), infections caused by multidrug-resistant (MDR) bacteria represent a significant challenge due to limited treatment options and high mortality rates. These bacteria, in fact, represent a significant threat as, using mechanisms such as enzymatic degradation, efflux pumps, and altered cell wall permeability, they make traditional treatments ineffective. Carbapenem resistance in Gram-negative bacteria typically results from the expression of antibiotic-inactivating enzymes and/or nonenzymatic mechanisms, often facilitated by horizontal transfer of carbapenemase genes through mobile genetic elements such as plasmids or transposons [1,2]. The socioeconomic impact of MDR bacterial infections is profound, resulting in increased healthcare costs, prolonged hospital stays, and reduced treatment options. This pressure on healthcare systems jeopardizes patient outcomes and quality of life [3,4,5,6].

Carbapenemase-producing Enterobacterales (CPEs) are classified based on the Ambler classification into class A (e.g., KPC), class B metallo-β-lactamases (e.g., NDM, VIM, IMP), and class D (e.g., OXA-48-like enzymes) [1]. These enzymes differ not only in their molecular structure but also in their resistance profiles and therapeutic implications. For example, KPC-producing strains may be susceptible to ceftazidime–avibactam, whereas this agent is ineffective against metallo-β-lactamases like NDM, which require alternative options such as aztreonam–avibactam or cefiderocol [2]. Understanding the type of carbapenemase involved is therefore critical for guiding appropriate therapy and infection control strategies. Against this background, our study focuses on NDM-producing strains, which are emerging in Italy and globally as a particularly challenging resistance mechanism in high-risk hospital settings.

In recent years, metallo-β-lactamase (MBL), including the New Delhi MBL (NDM) [7], an enzyme that not only confers significant antimicrobial resistance to bacteria, but also clinically impacts on infected patients, increasing the risk of mortality, has extended outside the Indian subcontinent. The scientific literature reports an increasing number of cases of infection and, in some circumstances, even epidemics throughout the world [8,9,10]. In Europe, particularly in the Balkan regions and Mediterranean areas, real outbreaks have occurred in countries like Spain, Portugal, Greece, and Italy [11,12,13]. In Italy the first detection dates to 2009, in a patient hospitalized in a bone marrow transplant unit [14], followed by several recent outbreaks of these extensively resistant phenotypes, with cases documented in regions like Tuscany [15,16,17,18], Lazio [19], and Lombardy [20]. Similarly to other infections caused by multiresistant bacteria, risk factors for NDM-*Enterobacterales* infections are a patient’s comorbidities, prolonged hospitalization or intensive care stay, mechanical ventilation, and dialysis, but also previous colonization and previous use of antimicrobials [21,22].

The identification of the carbapenemase type has an enormous clinical value, often underestimated given the rapid and irreversible possibility of worsening of patients in cases of invasive infection [23,24,25,26], as it directly informs the selection of effective antimicrobial therapy—particularly in the case of NDM production, which requires the use of specific agents such as aztreonam–avibactam or cefiderocol. In fact, therapeutic strategies in case of NDM-*Enterobacterales* infections require the use of specific antimicrobials [27,28,29,30,31,32] (the combination of Avibactam and Aztreonam [33], or Cefiderocol), mandatory for an effective therapeutic management.

The aim of the present study is to describe the local epidemiology, analyze possible risk factors linked to the development of invasive infections in colonized patients, and evaluate the impact of NDM infections on patient outcomes. Finally, although with limited data, the study can stimulate reflections on new therapeutic strategies and the development of possible further resistance [34,35].

To our knowledge, this is one of a few studies to explore the evolving resistance patterns and clinical burden of NDM-producing *Enterobacterales* over recent years in a high-risk ICU population in Northern Italy, providing insight into recent epidemiological shifts and local resistance profiles.

## 2. Methods

### 2.1. Study Design and Setting

The study is a single-center, retrospective, observational study conducted between 1 January and 31 August 2023 at the General and Cardiac Surgery Intensive Care Units of ‘Città della Salute e della Scienza’ University Hospital in Turin (Italy).

The study was designed in accordance with ethical principles for biomedical research involving human subjects, as outlined in the World Medical Association’s Declaration of Helsinki. Data collection occurred by anonymously applying the STROBE (Strengthening the Reporting of Observational Studies in Epidemiology) operational checklist [36]. The study was approved by the Interdepartmental Ethics Committee on 24 January 2020 with reference number 0008195 which authorized its exemption from obtaining individual consent given the observational study’s design and the envisaged anonymization procedures.

### 2.2. Population

All patients admitted to ICUs who showed colonization by *Enterobacterales* producing NDM during the study period, as confirmed by at least one surveillance sample (rectal swab, tracheal swab, urine cultures), were enrolled. Colonized patients were subsequently monitored for the development of invasive infections caused by NDM microorganisms, such as ventilator-associated pneumonia (VAP) or bloodstream infections (BSI).

Patients’ demographic characteristics, medical history, comorbidities, severity scores such as SAPS II (Simplified Acute Physiology Score II) and SOFA score (Sequential Organ Failure Assessment), use of organ supports during ICU admission such as mechanical ventilation, extracorporeal membrane oxygenation (ECMO) or renal replacement therapy (RRT) and duration if used, and laboratory and microbiological results were extracted from patients’ medical records, available in both paper and electronic formats. We considered an event as a previous infection only the infections occurred during hospital admission in the study period.

Infection control practices were specifically implemented and monitored during the study period, since the presence of clusters was identified; they adhered to standardized protocols in accordance with local and international recommendations for contact isolation, but differed from routine clinical practice in frequency as follows: (a) surveillance measures with an increase in microbiological samples (bi-weekly rectal swabs; rectal swabs at referral from ICU to other wards); (b) educational measures for healthcare staff and stakeholders; (c) hygienic–sanitary measures, in order to allow a more effective cohorting of cases and a limitation of the spread.

### 2.3. Outcomes

The primary outcome was 28-day mortality.

Patients were followed until hospital discharge to assess the following secondary outcomes:

Clinical course after antibiotic therapy at 3, 7, and 14 days, based on laboratory tests and patients’ clinical progress;

ICU, hospital, and 60-day mortality;

Length of ICU and hospital stay.

### 2.4. Definitions

In line with the surveillance protocol of the European Centre for Disease Control and Prevention (ECDC), an infection was defined as ‘acquired in intensive care’ if its signs and/or symptoms occurred at least 48 h after admission [37].

Bloodstream infection (BSI) was defined as positivity in at least one blood culture for a recognized pathogen or positivity in two blood cultures with a skin contaminant from two different blood samples, usually collected within 48 h, associated with the presence of at least one clinical symptom among the following: fever above 38 °C, chills, or hypotension [37]. Ventilator-associated pneumonia (VAP) was defined as follows: (1) two or more chest X-rays or CT scans suggestive of pneumonia in patients with underlying cardiac or pulmonary disease, or a definitive chest X-ray or CT scan in patients without underlying cardiac or pulmonary disease; (2) a fever higher than 38 °C and/or leukocytosis higher than or equal to 12,000 WBC/mm^3^ or leukopenia lower than or equal to 4000 WBC/mm^3^; (3) at least one of the following: (a) new purulent sputum or change in sputum characteristics; (b) cough, dyspnea, or tachycardia; (c) suggestive signs on auscultation (rales or bronchial breath sounds, rhonchi, wheezing); (d) worsening of gas exchange (e.g., desaturation, increased oxygen requirement, or increased ventilatory requirement) [37,38]. A hospital-acquired infection was defined as ‘associated with a medical device’ if there was use, even intermittent, of the particular device in the 48 h preceding the onset of the infection, including intubation, central vascular catheters, and urinary catheters [37].

The isolated microorganisms were defined as multiresistant (or MDR, multidrug-resistant) if they were resistant to at least one drug belonging to three or more different antimicrobial categories; they were defined as extensively drug-resistant (XDR) if sensitive to only one or two antimicrobial categories; they were defined as pan drug-resistant (PDR) if resistant to all antimicrobial agents in all categories [39].

In line with the latest definitions from the European Centre for Disease Control and Prevention [37,40], patients were defined as follows:

(1) Colonized—If the presence of a microorganism in equilibrium with the host system, without the appearance of signs and symptoms had been demonstrated;
(2) Infected—If physio-pathological alterations of the host, with consequent clinical signs and symptoms, were demonstrated.

Once a diagnosis of infection was established, patients were no longer considered part of the colonized group, even if they had previously been colonized.

We also considered possible carbapenemase-producing clusters. Epidemiological monitoring of potential clusters is routinary performed in the ‘Città della Salute e della Scienza’ university hospital in Turin. Clusters are defined as the presence of at least 3 patients simultaneously admitted to the same ward with positivity for the same Alert or MDR organism in a short period of time (30 days).

Appropriate treatment was defined as the administration of at least one in vitro active agent against NDM.

ICU-free days were calculated by subtracting the total ICU length of stay from 28 days. Patients who died within the 28-day period were assigned zero ICU-free days as a competitive event. Similarly, ventilator-free days (VFDs) were calculated by subtracting the total number of days on invasive mechanical ventilation from 28 days. Patients who died within the 28-day period were assigned zero VFDs as a competitive event.

### 2.5. Microbiological Monitoring, Typing and Susceptibility Assessment

Microbiological samples were collected according to local protocols. Surveillance cultures, including the execution of rectal swabs, tracheal aspirates, and urine cultures were obtained on a weekly basis, in the absence of clinical need. Further invasive and non-invasive microbiological investigations were performed based on clinical needs, according to the judgement of the attending physicians. Carbapenemase-producing *Enterobacterales* colonization status was assessed by surveillance rectal swabs collected according to local protocol: on patients’ ward admission, then every week and on patients’ discharge. Sampling was performed with ESwab (Copan, Brescia, Italy): 10 µL of transport medium was automatically seeded by the WaspLab platform (ADA, Padova, Italy) on Brilliance CRE agar (Thermo Fisher Scientific, Waltham, MA, USA) and incubated for 18–20 h at 35 ± 2 °C. The strains grown on the selective medium were identified and screened for carbapenemase production as reported below.

For blood cultures, blood was collected in BACT/ALERT FA Plus and BACT/ALERT FN Plus (Biomerieux, Marcy l’Étoile, France) bottles according to local protocol and incubated in BACT/ALERT^®^ VIRTUO (Biomerieux) for 5 days. Blood cultures flagged positive by the instrument were seeded on appropriate medium according to Gram-stain examination.

Microorganisms obtained from different samples (rectal, urinary, blood, respiratory, etc.) were identified by matrix-assisted laser desorption/ionization time-of-flight mass spectrometry (MALDI-TOF MS) Microflex LT (Bruker Daltonics GmbH & Co. KG, Bremen, Germany). Subsequently, significant pathogens isolated from clinical samples were examined to determine antimicrobial susceptibility testing (AST) using broth microdilution and the MicroScan^®^WalkAway^®^ 96 Plus system (Beckman Coulter, Brea, CA, USA). As regards cefiderocol AST, MDR strains were screened by the disc diffusion method and, in cases of area of technical uncertainty results, tested by broth microdilution with a ComASP Cefiderocol 0.008–128 µg/mL kit (Liofilchem, Roseto degli Abruzzi, Italy).

Antimicrobial susceptibility data were interpreted according to European Committee on Antimicrobial Susceptibility Testing (EUCAST) antibiotic breakpoints version 13, 2023 [41].

Carbapenemase-producing Enterobacterales (CPEs) were screened according to EUCAST rules on both clinical and surveillance positive cultures. CPEs and carbapenemase were confirmed by the Lateral Flow Immunoassay NG-Test CARBA 5 (NG Biotech, Guipry, France) immunochromatographic test and by the Xpert^®^ Carba-R (Cepheid, Sunnyvale, CA, USA) molecular test [42,43]. Enterobacterales-NDM strains were stored in cryogenic vials at −80 °C for subsequent clustering analysis.

### 2.6. Clustering Analysis

To investigate the potential difference between CPE-NDM colonizing and invasive strains, FT-IR spectroscopy (FTIRS) was used. Non-duplicated stored *Enterobacterales*-NDM strains, isolated from rectal swabs, colonizing strains, blood cultures, and invasive strains from infected patients, were seeded onto BD Columbia blood agar (Becton Dickinson, Franklin Lakes, NJ, USA) and incubated at 35 ± 2 °C for 18–20 h. The grown colonies were measured with the FTIRS-based IR Biotyper system (IRBT, Bruker Daltonics) in three independent biological replicates and five technical replicates according to the manufacturer’s instructions. Spectra were acquired and analyzed in transmission mode (spectral range: 1300–800 cm^−1^). Clustering analysis was performed by IR Biotyper Client Software v4.0 (Bruker Daltonics). The unsupervised dimensionality reduction method, principal component analysis (PCA), and linear discriminant analysis (LDA) were used to investigate the possibility of discriminating between colonizing and invasive strains. Each replicate spectrum was represented as a dot in the two-dimensional (2D) scatter plot.

### 2.7. Statistical Analysis

Descriptive data were tested for normal distribution by the Shapiro–Wilk test and presented as means and standard deviation (SD), medians and interquartile range (IQR) (continuous variables), or as numbers and percentages (categorical variables) as appropriate. The unpaired *t*-test or Wilcoxon rank-sum test were used for continuous variables as appropriate. Categorical variables were analyzed with the chi-square test or Fisher’s exact test, as appropriate. We assessed 28-day survival using the Kaplan–Meier survival estimate, and the log-rank test was used to analyze differences in the survival curves among groups (colonization and infection). The relationships between the ICU–free days and ventilator–free days and the variables under study were evaluated using the Spearman correlation coefficient or the Point Biserial correlation for categorical or dichotomous variables, respectively. A *p*-value <0.05 was considered statistically significant. All study variables were included as independent variables in an exact univariate logistic regression to assess their role as dependent variables in determining infection caused by *Enterobacterales* NDM. Multivariate exact logistic regression was performed using a selection of variables that showed statistical significance in univariate analysis (defined in this case as *p*-value < 0.05). Statistical analyses were conducted with Stata 16.1/SE (Stata Corp, TX, USA).

## 3. Results

### 3.1. Population Characteristics

A total of 915 patients were hospitalized during the study period (245 in the General Intensive Care Unit and 670 in the Cardiac Intensive Care Unit). Forty-six tested positive on a surveillance swab. Of these, 33 (72%) were colonized by a microorganism producing NDM-type carbapenemase, while 13 (28%) developed an invasive infection (Table 1). The colonization time was 4 days (IQR 2–11) for colonized and 9 days (IQR 6–14) for infected patients. Infection time was 16 days (IQR 8–31). The SAPS II score at ICU admission for the entire population was 40 ± 13. There was a trend towards a higher score in infected patients, but this did not reach statistical significance. No comorbidity was statistically significantly correlated with the development of infection, whereas dialysis (*p*-value 0.0280) and colonization time (*p*-value 0.0331) showed significant correlation. Previous general, particularly abdominal (*p*-value 0.045), and cardiac surgery were statistically significantly correlated with the development of infection (*p*-values of 0.018 and 0.026, respectively).

Among the 13 patients who developed an invasive infection, 2 had bacteremia (BSI), 10 had ventilator-associated pneumonia (VAP), and 1 had peritonitis. The antibiotic used as targeted therapy was Cefiderocol in six cases and Ceftazidime/Avibactam combined with Aztreonam in five cases. One patient received inappropriate empiric treatment; one patient was not treated because he died before diagnosis [Table 2].

### 3.2. Outcome

**Figure 1:**
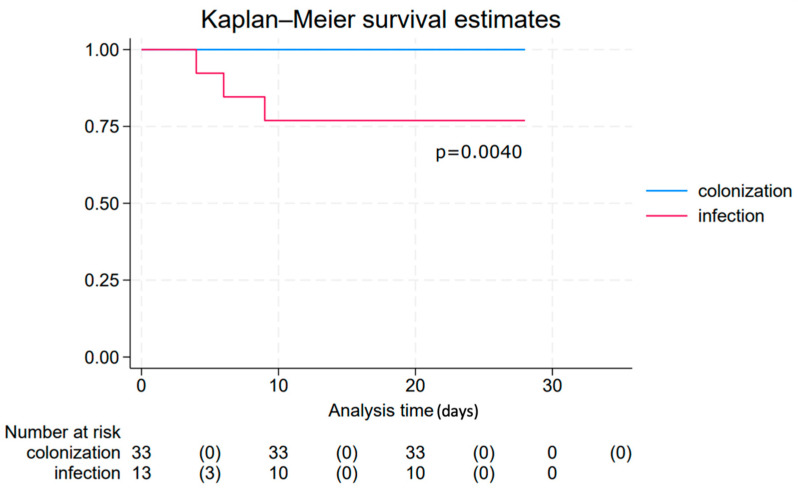
Kaplan–Meier survival estimates for the colonization and infection groups (total follow up time: 28 days). The event of interest was death. Time is plotted on the X-axis as days and probability of survival on the Y-axis.

Mortality among the 46 colonized and infected patients was 7% at 28 days and 9% at ICU discharge. All deaths occurred in the infected group, with three patients dying within 28 days and a total of four patients dying in intensive care; no deaths occurred in the colonized group (Figure 1). Hospital and ICU stay were significantly longer in the infected patients group (median 71 days, IQR 52–110; 39 days, IQR 22–58, respectively), compared to the colonized one (median 21 days, IQR 9–36; 7 days, IQR 3–21, respectively). The duration of mechanical ventilation was also significantly longer in infected patients (median of 28 days, IQR 18–37 vs. 1 day, IQR 1–5) (Table 1).

### 3.3. NDM-Infected Group

The characteristics of patients with invasive infection due to NDM microorganisms (*Klebsiella pneumoniae* in 10 and *Enterobacter cloacae* in 3 patients) are presented in Table 2. Of the 13 patients, 11 were men with high severity scores (SAPS II at admission of 48, IQR 36.0–53.5; SOFA at infection of 7, IQR 5.0–10.5) and 11 underwent surgery (4 major abdominal surgery; 7 cardiac surgery, of which 3 were transplants). The median time from ICU admission to colonization was 9 days (IQR 6–14), while the median time to infection was 16 days (IQR 8–31). Two patients were treated with empiric antibiotic therapy and eleven with targeted therapy, identified as cefiderocol or ceftazidime–avibactam in combination with aztreonam.

Combination therapy was mostly used with cefiderocol therapy (eight cases), while ceftazidime–avibactam + aztreonam was usually administered as monotherapy. The drugs used in combination were fosfomycin (three cases), gentamicin (five cases), tigecycline (one case). The details of the treatment are shown in Table 3. One patient was treated with other antibiotics on an empirical basis, and one was not treated, both of which had clinical failure (exitus) before the possibility of switching to targeted therapy. Susceptibility to cefiderocol was assessed in 11/13 patients, with susceptibility in 6 cases. Colistin sensitivity was not tested.

Considering the outcome of infected patients (Table 4), all patients treated with ceftazidime–avibactam and aztreonam showed clinical improvement at 7 and 14 days, with no deaths at 28 days or in the ICU. Among patients treated with other antibiotics, four patients (50%) reported an improvement at 7 days, and five (62%) at 14 days. The mortality at 28 days of this second group of patients is 50% (*p*-value > 0.05). The occurrence of infection and the duration of mechanical ventilation showed strong inverse correlations with ICU-free days (−0.62 and −0.85, respectively; *p* < 0.01). SAPS II at admission, cardiac surgery, dialysis, and previous infection moderately correlated with ICU-free days (−0.43, 0.40, −0.41, and −0.46, respectively; *p* < 0.01) (Table 5).

The exact logistic regression model highlighted that major surgery and renal replacement therapy were statistically associated with infection by NDM *Enterobacterales* in colonized patients at the univariate analysis. This association was confirmed through multivariate analysis (Table 6).

### 3.4. Surveillance and Infection Control Measures During the Study Period

**Figure 2:**
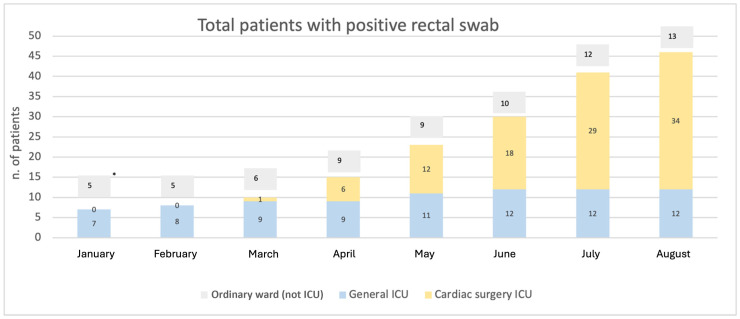
Total patients with positive rectal swab for NDM in the departments subject to surveillance in the period between January 2023 and August 2024. * indicates that this number refers to patients who developed an invasive infection during the observation time. ICU: Intensive Care Unit.

During the study period, a progressive rise in cases (intended as colonization/infection) of NDM *Enterobacterales* was observed, with increasing numbers in the wards under study. Figure 2 describes the trend of patients who tested positive for at least one microbiological sample, divided by observation ward; cases of invasive infection are specified above the columns in the figure.

**Figure 3:**
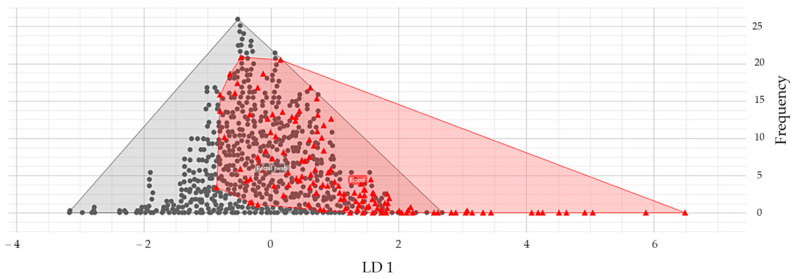
IR Biotyper^®^ Linear Discriminant Analysis results: Attempt to discriminate between *K. pneumoniae* colonizing and invasive strains. Grey circles: strains isolated from rectal swabs; red triangle: strains isolated from blood cultures.

As stated before, an NDM *Enterobacterales* cluster was defined as the presence of at least three patients simultaneously admitted to the same ward with positivity for this pathogen. In the study period between January and December 2023, in the General Intensive Care Unit, the presence of four possible clusters of CPEs of the NDM type and two clusters of CPE–KPC were reported. In the Cardiac Intensive Care Unit, the possible clusters were four of KPC and five of NDM. Forty-three strains from 35 (76.1%) enrolled patients positive for *K. pneumoniae* were selected for clustering analysis. A total of 645 spectra were acquired and analyzed. PCA and LDA dimension reduction analysis were applied with different principal components (PCs) and variance parameters to investigate colonizing and invasive strains in relationship to potential differences linked to virulence features. A substantial overlapping between the NDM *K. pneumoniae* colonizing and invasive strains was observed with both PCA and LDA. However, using LDA, which maximizes the intergroup variance and minimizes the intragroup variance, with 40 PCs and a 99.7% variance, a polar deviation of blood culture strains, more concentrated in the right area of the diagram, could be noted (Figure 3).

## 4. Discussion

The appearance of New Delhi-type carbapenemase (NDM) among Gram-negative bacteria represents a clinical threat of growing importance due to the rapid spread of highly drug-resistant clones [17] in Europe, and particularly in Italy [20,44]. The worldwide spread of MBL-carbapenemase-producing *Enterobacterales* infections is of great concern due to the implications in terms of reduced effective therapeutic options, increased mortality [22,23], and healthcare costs. However, specific data is lacking regarding the intensive care context and, in particular, on the different impacts of colonization and infection.

Data presented in our study represent the first data available in the Piedmont region, Italy, on NDM epidemiology and one of the largest datasets published so far. In fact, this study, conducted in the period between January and August 2023, confirms a rapid increase in cases of colonization by NDM-type *Enterobacterales* in the ICUs of our hospital, where a specific prospective project dedicated to the management of colonization and infection cases has been activated.

In the study period, out of a total of 915 patients (245 in the General ICU and 670 patients in the Cardiac ICU), 46 patients tested positive for colonization (surveillance swabs), equal to 5% of the total. These data appear lower than those detected by an Italian study in 2021, conducted on an entire university hospital in Pisa, which reported a positivity for any colonization by *Enterobacterales*-type pathogens equal to 16.4% of the surveillance swabs [15], with the presence of MDR in 12% of the rectal swabs performed upon admission to the hospital (9% of them represented by KPC- and NDM-type, the latter describing 41.4% of the cases).

These results were achieved thanks to an enormous surveillance effort aimed at the rapid identification of patients with colonization by carbapenemase-producing pathogens and their typing. In fact, since the presence of clusters in our ICUs was initially identified, the following have been implemented: (a) surveillance measures with an increase in microbiological samples (bi-weekly rectal swabs; rectal swabs at referral from ICU to other wards); (b) educational measures for healthcare staff and stakeholders; (c) hygienic–sanitary measures, in order to allow a more effective cohorting of cases and a limitation of the spread. The management of the outbreak required integrated multidisciplinary management between ICU healthcare staff, Health Management, Infectious Diseases consultants and Clinical Microbiologists. The effort made in this ongoing process was able to guarantee early interventions in response to the epidemiological trend, which was followed by important and positive clinical repercussions.

Among colonized patients (46), 13 (28%) developed an invasive infection by a microorganism producing NDM-type carbapenemase. This percentage is significantly higher than that described by H. Seo et al. [25] in a prolonged but not recent study (2010–2019) conducted in all departments in a Korean hospital. In that study, a percentage of 3% of patients colonized by NDM who acquire infection within 30 days is, in fact, reported. Indeed, it should be noted that most of the studies in the literature focus on infected patients only, with a lack of data on colonization [26], often outside the intensive care context, with, for example, a focus on geriatric patients [24] or specific objective of the microbiological analysis of possible clusters or resistance determinants [45].

In our population, no impact of comorbidities, even those present, on the development of infection emerged. Almost all patients (98%) were treated with invasive mechanical ventilation; 28% were dialyzed and 17% underwent heart and/or lung transplant. Dialysis was found to be statistically more used in the group of infected patients (*p* = 0.028). Half of the patients (47%) were affected by a previous invasive infection by any type of isolate with a greater, but non-statistically-significant (*p* = 0.098), distribution in the group of patients subsequently infected by CPE–NDM and/or who had undergone previous treatment with meropenem (*p* = 0.087). Infection did not appear to be more frequent in the subgroup undergoing major non-cardiac surgery (*p* = 0.018), while cardiac surgery appeared to be more frequent in the group of colonized patients (*p* = 0.026), perhaps due to the shorter post-operative course.

Colonization manifested itself after an average time of 6 (3–11) days from admission to the ICU, lower than in the comparative literature [23], probably due to the high ecological pressure as discussed above. The infection time was instead significantly longer, equal to 16 (8–31) days, in line with the literature [23]. It can be hypothesized that this reflects not only the need for more time to reach an adequate load to determine infection, but also the longer stay in the ICU of critically ill patients in highly fragile conditions.

Our cohort was characterized by an overall reduced mortality (28-day mortality 7%, ICU mortality 9%), with no deaths in the group of colonized patients. However, it emerges that both 28-day mortality (*p* = 0.019) and intensive care mortality (*p* = 0.004) are higher in the infected group. Mortality in the infected group (38% at 28 days, 50% in intensive care) is therefore close to that reported by Falcone et al. (29.7%) in all hospital wards and not only in the Intensive Care Unit [26]. Of note, among patients treated with targeted therapy, all patients treated with CZA + ATM showed clinical improvement at 7 and 14 days, with no ICU mortality at 28 days. The small number of treatment subgroups and the presence of only one death among patients treated with compatible antimicrobials do not allow further evaluation.

Regardless of the small sample size, the analysis of the correlation with the length of stay in the ICU highlighted that the presence of infection, a higher SAPSII at admission, chronic lung disease, non-cardiac surgery, dialysis treatment, previous infection, and previous treatment with meropenem and with ceftazidime/avibactam all tended to increase ICU length of stay with weak but significant strength.

The multivariate analysis designed to identify the determinants of infection among colonized patients, although conditioned by the low mortality of our cohort, identified dialysis (OR 8.2461, *p* = 0.0173, CI95% [1.3636–65.9114]) and surgery (OR 22.8747, *p* = 0.0149, CI95% [1.5986–1447.743]) as factors that may impact on the risk of developing infection. Other variables considered—even those apparently more correlated—did not show statistical significance.

Considering the 13 patients with infection, they are characterized by high severity scores (SAPS II at admission of 48, IQR 36.0–53.5; SOFA at infection of 7, IQR 5.0–10.5); during hospitalization, 11 patients out of 13 underwent surgery.

Eleven patients were treated with targeted antibiotic therapy. According to the literature, the combination of ceftazidime–avibactam (CZA) and aztreonam (ATM) has been associated with a reduced risk of mortality compared to colistin-containing regimens [32] and is currently recommended as a first-line option against MBL [29,30,31,32]. In our context, six patients were treated with cefiderocol, usually as combination therapy, of which five improved at 7 and 14 days from the onset of infection, while one died within 7 days of the diagnosis of infection. In another five patients, ceftazidime–avibactam plus aztreonam (CZA + ATM) was used. In two cases, the identification of the carbapenemase subtype occurred after the first empirical therapy was initiated, which subsequently resulted in the treatment being inappropriate, with fatal outcomes.

We further performed cluster analysis since data from the literature support that, in addition to patients’ features, comorbidities at the onset of invasive forms of *K. pneumoniae* infections could be linked to colonization by strains with a particular virulence pattern [20]. In our study, to investigate this aspect, FT-IRS, a promising technique for fast clustering analysis, strain typing, and subspecies discrimination was used. Unfortunately, in our analysis, both PCA and LDA could not discriminate between colonizing and invasive strains, with no clear contribution to the explanation of the occurrence of invasive infections in a limited subgroup of patients. However, these results are based on the comparison of only eight patients with positive blood cultures, probably belonging to three different clusters. To better understand the role of FTIRS and its real potentialities strain virulence discrimination, a next-generation sequencing approach should be combined.

Being able to identify the carbapenemase subtype becomes essential for an adequate therapeutic strategy, and the colonization data themselves must be specified, to avoid cross-contamination and adequate cohorting. This aspect is even more true in critical areas, such as ICUs, where often the criticality of the patients and the onset of refractory septic shock conditions do not allow traditional diagnostic turn-around times.

In conclusion, the high prevalence of NDM-producing Enterobacterales and their extensive resistance patterns underline the importance of early detection and appropriate empirical antimicrobial strategies in critically ill patients. These findings emphasize the role of local epidemiological surveillance in guiding therapeutic decisions and support the integration of targeted agents such as aztreonam–avibactam or cefiderocol into clinical practice. Incorporating such evidence into antimicrobial stewardship efforts is crucial to improve patient outcomes and curb the further spread of resistance.

## 5. Limits

The study has some limitations. First, it is a monocentric study on a cohort of critically ill patients; therefore, the data may not be clearly representative of larger populations in different contexts. Furthermore, a complete comparison with the population not colonized nor infected by NDM *Enterobacterales*, which would have allowed confirmation of its role in terms of risk factors and impact on the severity of the disease and on mortality, has not been carried out. Since this is an observational study, variations in the management and implementation of infection control measures may have influenced the distribution of cases, as well as the training of the healthcare staff involved and the modification of some management and logistical aspects. The number of invasive infections was relatively low, as was the number of deaths, and this limited the possibility of enhancing the multivariate logistic regression model’s statistical power, although it allowed us to identify some potentially interesting correlations worthy of future investigation. The logistic regression model might thus be saturated, especially given the limited number of events. The low number of infected patients did not allow us to delve deeper into the impact of the new antimicrobials used.

## 6. Conclusions

This monocentric observational study underscores the epidemiological impact of colonization and infection by NDM-producing carbapenemase-producing Enterobacterales (CPEs) in an ICU setting in a university hospital in Italy. Our findings highlight the urgent need to implement targeted surveillance strategies for emerging resistance mechanisms, particularly in case of possible new clusters. While infection control measures remain a cornerstone in managing multidrug-resistant organisms, our results emphasize that timely and accurate identification of the specific carbapenemase type is crucial, particularly in critically ill patients. This is especially relevant given the limited—but potentially effective—treatment options available for NDM-producing *Enterobacterales*. Finally, further prospective, multicenter studies are warranted to better characterize the epidemiological trends and to optimize therapeutic approaches.

## Figures and Tables

**Table 1:** Population characteristics.

Variable (*n*; %), Mean ± SD, or Median (IQR)	Total (n = 46)	Colonized (n = 33)	Infected (n = 13)	*p*-Value
Male sex	36 (78)	25 (76)	11 (85)	
Age	63 (IQR 54–71)	63 (IQR 54–70)	57 (IQR 52–72)	
BMI	26.3 (IQR 22.0–29.3)	24.4 (IQR 21.5–28.7)	28.3 (IQR 26.0–29.7)	
**Comorbidities**				
Active smoking	6 (13)	5 (15)	1 (8)	0.659
History of smoking	13 (28)	9 (27)	4 (31)	1.000
Alcohol/drug abuse	2 (4)	1 (3)	1 (8)	0.490
Cardiovascular disease	30 (65)	22 (67)	8 (62)	0.774
Hypertension	14 (30)	10 (30)	4 (31)	1.000
Respiratory disease	14 (30)	8 (24)	6 (46)	0.171
Renal failure	8 (17)	4 (12)	4 (31)	0.196
Hepatic failure	0	0	0	ND
Chronic neurological disease	3 (7)	1 (3)	2 (15)	0.188
Active solid malignancy	3 (7)	2 (6)	1 (8)	1.000
Previous solid malignancy	5 (11)	4 (12)	1 (8)	1.000
Active hematological malignancy	2 (4)	1 (3)	1 (8)	0.490
Previous hematological malignancy	1 (2)	0 (0)	1 (8)	0.283
Diabetes mellitus	7 (15)	5 (15)	2 (15)	1.000
**Clinical characteristics and treatments**				
SAPS II at admission	40 ± 13	38 ± 12	45 ± 13	0.1480
ECMO	2 (4)	1 (3)	1 (8)	0.490
RRT	13 (28)	6 (18)	7 (54)	0.028
Mechanical ventilation	45 (98)	32 (97)	13 (100)	1.000
Previous infection	21 (47)	12 (40)	9 (70)	0.098
Previous fungal infection	3 (7)	2 (6)	1 (8)	1.000
Time to colonization	6 (3–11)	4 (2–11)	9 (6–14)	0.0331
Previous meropenem treatment	7 (15)	3 (9)	4 (31)	0.087
Previous ceftazidime–avibactam treatment	5 (11)	2 (6)	3 (23)	0.128
Time to infection			16 (8–31)	
**Diagnosis at ICU admission**				
Sepsis/septic shock	9 (20)	4 (12)	5 (39)	0.092
Major non-cardiac surgery	5 (11)	1 (3)	4 (31)	0.018
Cardiac surgery	35 (76)	28 (85)	7 (54)	0.026
Heart/pulmonary transplant	8 (17)	5 (15)	3 (23)	0.669
Acute respiratory failure	3 (7)	2 (6)	1 (8)	1.000
Cardiogenic shock	4 (9)	3 (9)	1 (8)	1.000
**Type of surgery**				
Abdominal surgery	6 (13)	2 (6)	4 (31)	0.045
Elective valve replacement	11 (24)	10 (30)	1 (8)	0.141
CABG	1 (2)	1 (3)	0 (0)	1.000
Ventricular assist device implant	2 (4)	1 (3)	1 (8)	0.490
Emergency cardiac surgery	12 (27)	10 (30)	2 (17)	0.461
Cardiac transplant	3 (7)	2 (6)	1 (8)	1.000
Lung transplant	4 (9)	2 (6)	2 (15)	0.565
Heart–lung transplant	1 (2)	1 (3)	0 (0)	1.000
**Outcome**				
MV days	3 (1–23)	1 (1–5)	28 (18–37)	0.0002
Ventilator-free days	25 (0–27)	27 (23–27)	0 (0–0)	0.0000
ICU mortality	4 (9)	0 (0)	4 (31)	0.004
28-day mortality	3 (7)	0 (0)	3 (23)	0.019
ICU length of stay	16 (4–39)	7 (3–21)	39 (22–58)	0.0003
ICU-free days	10 (0–24)	21 (7–25)	0 (0–0)	0.0000
Hospital length of stay	26 (12–62)	21 (9–36)	71 (52–110)	0.0029
**Site of infection**				
BSI	-	-	2 (15)	
VAP	-	-	10 (77)	
Peritonitis	-	-	1 (8)	
**Treatment**				
Cefiderocol	-	-	6 (46)	
CZA	-	-	5 (38)	
No treatment/inappropriate treatment	-	-	2 (16)	
**Infected outcome**				
Improvement in 7 days	-	-	9 (69)	
Improvement in 14 days	-	-	10 (77)	

List of abbreviations: BMI: body mass index; ECMO: extracorporeal membrane oxygenation; CABG: coronary artery bypass graft; RRT: renal replacement therapy; MV: mechanical ventilation; BSI bloodstream infection; VAP: ventilation-associated pneumonia; ICU: Intensive Care Unit; CZA: Ceftazidime–Avibactam/Aztreonam.

**Table 2:** NDM infected patients’ clinical characteristics, treatment strategies, and outcomes.

	Age, Sex	Diagnosis on Admission	SAPSII	Time to Colonization	Time to Infection	Site	Pathogen	SOFA at Infection	Treatment (Backbone ATB)	Choice of Antibiotic *	Outcome at 7 Days °	ICU Mortality	28-Day Mortality
1	86, M	Sepsis/septic shock	41	6	8	VAP	Kp	6	Meropenem/Vaborbactam	E	1	1	1
2	49, M	Orthotopic heart transplant	26	16	17	VAP	Kp	10	Avibactam/Aztreonam	T	0	0	0
3	63, M	Endocarditis	65	9	14	BSI	Ec	12	No treatment	T	1	1	1
4	57, M	Orthotopic lung transplant	31	3	4	BSI	Kp	3	Avibactam/Aztreonam	T	0	0	0
5	65, F	Acute respiratory failure	46	6	8	VAP	Kp	11	Cefiderocol	T	1	1	1
6	19, M	Orthotopic lung transplant	19	14	16	VAP	Ec	6	Avibactam/Aztreonam	T	0	0	0
7	72, M	Sepsis/septic shock	51	37	49	VAP + BSI	Kp	4	Cefiderocol	T	0	0	0
8	52, M	Aortic dissection type A	48	10	41	VAP + BSI	Kp	9	Cefiderocol	T	0	0	0
9	55, M	Sepsis/septic shock	56	3	33	VAP + BSI	Kp	4	Cefiderocol	E	0	0	0
10	22, F	Sepsis/septic shock	50	6	8	VAP + BSI	Kp	6	Cefiderocol	T	0	0	0
11	73, M	Sepsis/septic shock	48	19	19	Peritonitis + BSI	Kp	13	Cefiderocol	T	0	1	0
12	55, M	Cardiogenic Shock	41	11	31	VAP + BSI	Ec	7	Avibactam/Aztreonam	T	0	0	0
13	73, M	Elective cardiac surgery	58	8	15	VAP + BSI	Kp	10	Avibactam/Aztreonam	T	0	0	0

List of abbreviations: SAPSII: Simplified Acute Physiology Score II; M: male; F: female; VAP: ventilator-associated pneumonia; BSI: bloodstream infection; Kp: Klebsiella pneumona; Ec: Enterobacter cloacae; * = Antibiotic choice: E: empiric treatment; T: target treatment; ° = outcome at seven days: 0: alive; 1: dead.

**Table 3:** Treatment of infected population. R: resistant; S: sensitive.

Patient	Backbone Therapy	Combination Therapy	Cefiderocol Susceptibility	Ceftazidime/Avibactam Susceptibility	Therapy Duration
1	Meropenem/Vaborbactam	Fosfomycin	-	R	3
2	Avibactam/ Aztreonam	-	R	R	14
3	-	-	R	R	-
4	Avibactam/ Aztreonam	Fosfomycin	S	R	13
5	Cefiderocol	Fosfomycin/ Gentamicin	S	R	2
6	Avibactam/ Aztreonam	-	-	-	7
7	Cefiderocol	Gentamicin	S	R	17
8	Cefiderocol	Gentamicin	S	R	9
9	Cefiderocol	Fosfomycin	S	R	-
10	Cefiderocol	Gentamicin	S	R	12
11	Cefiderocol	Gentamicin/ Tygecycline	R	R	13
12	Avibactam/ Aztreonam	-	R	R	6
13	Avibactam/ Aztreonam	-	R	R	7

**Table 4:** Infected patients’ outcomes. ICU: Intensive Care Unit.

Variable (n; %)	Total (n = 13)	Ceftazidime-Avibactam + Aztreonam (n = 5)	Other (n = 8)	*p*-Value
Outcome				
Improvement in 7 days	9 (69)	5 (100)	4 (50)	0.105
Improvement in 14 days	10 (77)	5 (100)	5 (63)	0.231
ICU mortality	4 (31)	0 (0)	4 (50)	0.105
28-day mortality	3 (23)	0 (0)	3 (38)	0.231
Ventilator-free days	0 (0–0)	5 (0–9)	0 (0–0)	0.0175
ICU-free days	0 (0–0)	0 (0–0)	0 (0–0)	0.2059

**Table 5:** Spearman or Point Biserial correlation coefficients between study variables and ICU-free days. BMI: body mass index.

Dependent Variable	Independent Variable	Spearman’s Test	
		*Rho/pbis*	*p*-Value
ICU-free days	Infection (vs. colonization)	−0.6207	**0.0001**
	Age	0.2298	0.1241
	BMI	0.0261	0.8629
	SAPS II at admission	−0.4275	**0.0037**
	MV days	−0.8533	0.0000
	Septic shock	−0.4356	0.0025
	Transplant	−01907	0.2044
	Acute kidney injury	−0.0866	0.5671
	Cardiogenic shock	0.1356	0.3689
	Lung transplant	−0.1012	0.5034
	Heart transplant	−0.0866	0.5671
	CZA	0.3508	0.2399
	Active smoking	−0.0251	0.8685
	History of smoking	0.2133	0.1546
	Alcohol abuse	−0.0419	0.7824
	Cardiopathy	0.0753	0.6187
	Arterial hypertension	0.0648	0.6686
	Chronic lung disease	−0.2914	**0.0494**
	Renal insufficiency	−0.1756	0.2431
	Chronic neurological disorder	−0.2719	0.0676
	Active solid malignancy	0.1218	0.4199
	Previous solid malignancy	0.1182	0.4339
	Active hematological malignancy	0.0049	0.9743
	Previous hematological malignancy	−0.1534	0.3086
	Diabetes mellitus	0.0097	0.9490
	Major surgery	−0.3595	**0.0141**
	Cardiac surgery	0.4028	**0.0055**
	Haemodialysis	−0.4132	**0.0043**
	ECMO	−0.2195	0.1428
	Previous meropenem	−0.2981	**0.0442**
	Previous CZA	−0.3595	**0.0141**
	Previous infection	−0.4598	**0.0005**
	Previous fungal infection	−0.2224	0.1420

Ceftazidime/Aztreonam; ECMO: extracorporeal membrane oxygenation; ICU: Intensive Care Unit; MV: mechanical ventilation; SAPS: Simplified Acute Physiology Score. Bold = statistical significance.

**Table 6:** Univariate and multivariate logistic regression analysis of risk factors for development of NDM infection in colonized patients.

Dependent Variable	Independent Variable	Univariate Analysis			Multivariate Analysis		
		OR	*p*-Value	IC 95%	OR	*p*-Value	IC 95%
Infection	Age	0.9779	0.3005	0.9360–1.0204			
	BMI	1.0625	0.3571	0.9351–1.2141			
	SAPS II at admission	1.0401	0.1533	0.9860–1.1012			
	Septic shock	4.3536	0.1143	0.7462–27.8371			
	Transplant	1.6598	0.8057	0.2179–10.5064			
	Acute respiratory failure	1.2842	1.0000	0.0202–26.8678			
	Cardiogenic Shock	0.8365	1.0000	0.0147–11.6711			
	Lung transplant	2.7455	0.6292	0.1790–42.2145			
	Heart transplant	1.2842	1.0000	0.0202–26.8678			
	Transplant	2.5385	1.0000	0–99			
	Active smoking	0.4734	0.8953	0.0091–4.9315			
	History of smoking	1.1807	1.0000	0.2115–5.7297			
	Alcohol abuse	2.6018	0.9797	0.0314–215.7238			
	Cardiopathy	0.8040	0.9992	0.1778–3.9043			
	Arterial hypertension	1.0217	1.0000	0.1852–4.8552			
	Chronic lung disease	2.6161	0.2732	0.5525–12.5425			
	Renal insufficiency	3.1279	0.2855	0.4799–20.7287			
	Chronic neurological disorder	5.5558	0.3768	0.2654–353.6526			
	Active solid malignancy	1.2842	1.0000	0.0202–26.8678			
	Previous solid malignancy	0.6102	1.0000	0.0113–7.0632			
	Active hematological malignancy	2.6018	0.9797	0.0314–215.7238			
	Previous hematological malignancy	2.5385	0.5652	0.0650–+inf			
	Diabetes mellitus	1.0178	1.0000	0.0849–7.4558			
	Major surgery	13.2201	0.0363	1.1318–721.3683	22.8747	0.0149	1.5986–1447.743
	Cardiac surgery	0.2172	0.0727	0.0386–1.1258			
	Haemodialysis	5.0247	0.0440	1.0379–26.7039	8.2461	0.0173	1.3636–65.9114
	ECMO	2.6018	0.9797	0.0314–215.7238			
	Previous meropenem	4.2726	0.1733	0.6022–34.9169			
	Previous CZA	4.4651	0.2566	0.4458–60.6689			
	Previous infection	3.6361	0.1080	0.7978–19.9066			
	Previous fungal infection	1.2436	1.0000	0.0196–26.0385			
	Admission at General ICU	4.6047	0.0727	0.8882–25.8871			

BMI, body mass index; CZA, Ceftazidime/Aztreonam; ECMO, Extra Corporeal Membrane Oxygenator; ICU, Intensive Care Unit; SAPS II, Simplified Acute Physiology Score.

## Data Availability

The datasets used and/or analyzed in the current study are available from the corresponding author upon reasonable request.

## Abbreviations

NDM, New Delhi Metallo-beta-lactamase; ICU, Intensive Care Unit; SAPS II, Simplified Acute Physiology Score; MDR, multidrug-resistant; MBL, metallo-β-lactamase; VAP, ventilator-associated pneumonia; BSI, bloodstream infections; ECMO, extracorporeal membrane oxygenation; RRT, renal replacement therapy; ECDC, European Centre for Disease Control and Prevention; XDR, extensively drug-resistant; PDR, pan drug-resistant; VFDs, ventilator-free days; EUCAST, European Committee on Antimicrobial Susceptibility Testing; CPEs, Carbapenemase-producing *Enterobacterales*; PCA, principal component analysis; LDA, linear discriminant analysis; SD, standard deviation; IQR, interquartile range; BMI: body mass index; CABG: coronary artery bypass graft; MV: mechanical ventilation; CZA: Ceftazidime/Aztreonam.
